# Supplementary material for: Analysis of PPARγ Signaling Activity in Psoriasis
Source: Int J Mol Sci. 2021 Aug 10;22(16):8603. doi: 10.3390/ijms22168603 (PMC8395241; doi:10.3390/ijms22168603)
Supplement: Supplementary file 1 [file ijms-22-08603-s001.zip › Supplemental materials_Analysis of PPARg signaling activity in psoriasis/Pathway models/Models images and html files/Model of PPARG related pathways in psoriasis (short version)/6308.html]

IL1A


# Protein IL1A

|  |  |
| --- | --- |
| URN | urn:agi-llid:3552 |
| Total Entities | 0 |
| Connectivity | 5556 |
| Name | IL1A |
| Description | interleukin 1, alpha |
| Notes | The protein encoded by this gene is a member of the interleukin 1 cytokine family. This cytokine is a pleiotropic cytokine involved in various immune responses, inflammatory processes, and hematopoiesis. This cytokine is produced by monocytes and macrophages as a proprotein, which is proteolytically processed and released in response to cell injury, and thus induces apoptosis. This gene and eight other interleukin 1 family genes form a cytokine gene cluster on chromosome 2. It has been suggested that the polymorphism of these genes is associated with rheumatoid arthritis and Alzheimer's disease. [provided by RefSeq, Jul 2008] |
| Primary Cell Localization | Extracellular |
| Class | Ligand |
| ObjectType | Protein |

---

|  |  |
| --- | --- |
| Pathway | Plasmin Effects in Inflammation |
|  | Bradykinin Effects in Inflammation |
|  | Toll-like Receptors in Sterile Inflammation |
|  | Toll-like Receptor Independent Sterile Inflammation |
|  | Hypothalamic-Pituitary-Adrenal Axis in Acute Phase Response |
|  | Histamine Effects in Inflammation |
|  | Eosinophil Activation |
|  | Mast-Cell Activation without Degranulation |
|  | Mast-Cells De Novo Synthesized Mediators via IgE Independent Signaling |
|  | Mast-Cell Activation without Degranulation through IL1R1 and TLR Signaling |
|  | Negative Acute Phase Proteins Synthesis |
|  | Positive Acute Phase Proteins Synthesis |
|  | Proteins Involved in Atherosclerosis |
|  | Proteins with Altered Expression in Acne Vulgaris |
|  | Proteins Involved in Acne Vulgaris |
|  | Keratinocyte Proliferation in Acne Vulgaris |
|  | Apoptotic Keratinocytes Clearance Recession in Systemic Lupus Erythematosus |
|  | Dendritic Cell Activation in Systemic Lupus Erythematosis |
|  | Tubular Cell Dysfunction in Progressive Diabetic Nephropathy |
|  | Endothelial Cell Dysfunction in Early Diabetic Nephropathy |
|  | Proteins Involved in Diabetic Nephropathy |
|  | Microangiopathy in Diabetic Neuropathy |
|  | Hepatic Stellate Cells in non-Alcoholic Fatty Liver Disease |
|  | Proteins Involved in non-Alcoholic Fatty Liver Disease |
|  | Proteins Involved in Graves Disease |
|  | Graves Ophthalmopathy |
|  | Thymic Follicular Hyperplasia |
|  | Proteins Involved in Obesity |
|  | Antiphospholipid Antibodies in Endothelial Cell |
|  | TNFR/NF-kB Classical Pathway in Multiple Myeloma Plasma Cells |
|  | TNFR/NF-kB Alternative Pathway in Multiple Myeloma Plasma Cells |
|  | Antiphospholipid Antibodies and Endothelial Cell Interaction |
|  | Proteins Involved in Osteoarthritis |
|  | Osteoclast Activation in Psoriatic Arthritis |
|  | Proteins Involved in Rheumatoid Arthritis |
|  | Autocrine Cytokine/Chemokine Loops in Systemic Scleroderma |
|  | Genes with Mutations Associated with Systemic Scleroderma |
|  | Proteins Involved in Systemic Scleroderma |
|  | Proteins Involved in Periodontitis |
|  | IL1 Receptor Antagonist (IL1RN) Deficiency (DIRA) |
|  | IL36 Receptor Antagonist (IL1F5) Deficiency (DITRA) |
|  | Proteins with Altered Expression in Psoriatic Arthritis |
|  | Microglia Activation in Alzheimer's Disease |
|  | Proteins Involved in Glioblastoma |
|  | Proteins Involved in Glioma |
|  | Microglia Activation in Parkinson's Disease |
|  | MBP/MOG/PLP in Immune System Activation |
|  | Proteins Involved in Endometriosis |
|  | Proteins Involved in Spontaneous Abortion |
|  | Proteins Involved in Cholesteatoma |
|  | Subepithelial Fibroblasts in Middle Ear Cholesteatoma |
|  | HPV E6 and E7 Induced Disruption of TNFR/NF-kB Signaling in Keratinocytes |
|  | Cytokines Trigger Otitis Media |
|  | Alveolar Macrophage Negative Regulation Declined in COPD |
|  | Alveolar Macrophages Dysfunction in COPD |
|  | Plasmin Role in Glomerulonephritis |
|  | Endothelial Cell Dysfunction in Glomerulonephritis |
|  | AREG -> AP-1 Expression Targets |
|  | Noradrenaline/Gs Expression Targets |
|  | Noreadrenaline/Gq Expression Targets |
|  | CXCL12 Expression Targets |
|  | EGF -> CTNN Expression Targets |
|  | EGF -> AP-1/ATF Expression Targets |
|  | EGF -> CREB/CREBBP/ELK/SRF/MYC Expression Targets |
|  | EGF -> MEF/MYOD/NFATC Expression Targets |
|  | EGF -> STAT Expression Targets |
|  | F2 -> AP-1/CREB/ELK/SRF/SP1 Expression Targets |
|  | F2 -> STAT1/NF-kB Expression Targets |
|  | PLG -> AP-1/CREB/ELK/SRF/SP1 Expression Targets |
|  | PLG -> STAT1/NF-kB Expression Targets |
|  | GH1/GHR -> NF-kB/ELK/SRF/MYC Expression Targets |
|  | GH1/GHR -> STAT Expression Targets |
|  | GH1/PRLR Expression Targets |
|  | NFKB Canonical Signaling Activation in Cancer |
|  | NFKB Signaling Activation by Blocking of Tumor Suppressors |
|  | VEGFA Dependent Angiogenesis in Cancer |
|  | EGFR -> Expression Targets in Skin |
|  | IFNG/IFNR Expression Targets |
|  | IL1R -> STAT3 Signaling |
|  | IL1R -> NF-kB Signaling |
|  | IL2 Expression Targets |
|  | PAF/Gq -> AP-1/ATF1/CREB/ERK/SRF Expression Targets |
|  | CD Markers -> Expression Targets in Lymphoid System and Blood |
|  | PRL/PRLR Expression Targets |
|  | PRL/GHR -> STAT Expression Targets |
|  | PRL/GHR -> NF/kB/ELK/SRF/MYC Expression Targets |
|  | KITLG -> STAT Expression Targets |
|  | KITLG -> AP-1/CREB/CREBBP/MYC Expression Targets |
|  | PAF Expression Targets |
|  | NF-kB Canonical Signaling |
|  | TGFA -> STAT Expression Targets |
|  | TGFA -> CTNNB/CTNND Expression Targets |
|  | TGFA/AP-1/ATF Expression Targets |
|  | TGFA -> CREB/CREBBP/ELK-SRF/MYC Expression Targets |
|  | FASLG Expression Targets |
|  | TNF -> NF-kB Expression Targets |
|  | TNFSF10 Expression Targets |
|  | TNF -> AP-1 Expression Targets |
|  | CD72 -> AP-1 Expression Targets |
|  | IL1B -> PGE2 Expression Targets |
|  | IL1B Expression Targets |
|  | TLR4 -> NF-kB/IRF Expression Targets |
|  | IL1A Expression Targets |
|  | prarg negative regulators, ps-positive |
|  | prarg neg,ukn expres targets, ps-positive |
|  | PPARG negative regulators |
|  | PPARG negative targets |
|  | pparg expr target\_regulators |
|  | Neighbors of disease exacerbation |
|  | Neighbors of tumor progression |
|  | Neighbors of cancer progression |
|  | Neighbors of inflammatory status |
|  | Neighbors of overall survival |
|  | Neighbors of disease severity |
|  | Neighbors of tumor size |
|  | Neighbors of mortality |
|  | Neighbors of survival rate |
|  | Neighbors of infarct size |
|  | Neighbors of survival time |
|  | Neighbors of functional recovery |
|  | Neighbors of bacterial load |
|  | Neighbors of cancer survival |
|  | Neighbors of therapeutic efficacy |
|  | Neighbors of lethality |
|  | Neighbors of tumor regression |
|  | Neighbors of tumor response |
|  | Neighbors of treatment outcome |
|  | Neighbors of bone mass |
|  | Neighbors of body weight |
|  | Neighbors of body weight gain |
|  | Neighbors of cardiovascular risk |
|  | Neighbors of tumor promotion |
|  | Neighbors of neutrophil count |
|  | Neighbors of morbidity |
|  | Neighbors of bone mineral density |
|  | Neighbors of radioresistance |
|  | Neighbors of cell density |
|  | Neighbors of viral load |
|  | Neighbors of blood pressure |
|  | Neighbors of leukocyte count |
|  | Neighbors of vascular leakage |
|  | Neighbors of muscle mass |
|  | Neighbors of cancer incidence |
|  | Neighbors of blood glucose |
|  | Neighbors of osteoclast number |
|  | Neighbors of energy expenditure |
|  | Neighbors of breast cancer risk |
|  | Neighbors of cell permeability |
|  | Neighbors of fertility |
|  | Neighbors of lymphocyte count |
|  | Neighbors of monocyte count |
|  | Neighbors of metabolic response |
|  | Neighbors of bone volume |
|  | Neighbors of fibroblast phenotype |
|  | Neighbors of fractional shortening |
|  | Neighbors of disease exacerbation |
|  | Neighbors of tumor progression |
|  | Neighbors of cancer progression |
|  | Neighbors of inflammatory status |
|  | Neighbors of overall survival |
|  | Neighbors of disease severity |
|  | Neighbors of tumor size |
|  | Neighbors of mortality |
|  | Neighbors of survival rate |
|  | Neighbors of infarct size |
|  | Neighbors of survival time |
|  | Neighbors of functional recovery |
|  | Neighbors of bacterial load |
|  | Neighbors of cancer survival |
|  | Neighbors of therapeutic efficacy |
|  | Neighbors of lethality |
|  | Neighbors of tumor regression |
|  | Neighbors of tumor response |
|  | Neighbors of treatment outcome |
|  | Neighbors of bone mass |
|  | Neighbors of body weight |
|  | Neighbors of body weight gain |
|  | Neighbors of cardiovascular risk |
|  | Neighbors of tumor promotion |
|  | Neighbors of neutrophil count |
|  | Neighbors of morbidity |
|  | Neighbors of bone mineral density |
|  | Neighbors of radioresistance |
|  | Neighbors of cell density |
|  | Neighbors of viral load |
|  | Neighbors of blood pressure |
|  | Neighbors of leukocyte count |
|  | Neighbors of vascular leakage |
|  | Neighbors of muscle mass |
|  | Neighbors of cancer incidence |
|  | Neighbors of blood glucose |
|  | Neighbors of osteoclast number |
|  | Neighbors of energy expenditure |
|  | Neighbors of breast cancer risk |
|  | Neighbors of cell permeability |
|  | Neighbors of fertility |
|  | Neighbors of lymphocyte count |
|  | Neighbors of monocyte count |
|  | Neighbors of metabolic response |
|  | Neighbors of bone volume |
|  | Neighbors of fibroblast phenotype |
|  | Neighbors of fractional shortening |
|  | Neighbors of cell development |
|  | Neighbors of immune response |
|  | Neighbors of cell population |
|  | Neighbors of T-cell development |
|  | Neighbors of inflammatory response |
|  | Neighbors of wound healing |
|  | Neighbors of angiogenesis |
|  | Neighbors of innate immune response |
|  | Neighbors of cell function |
|  | Neighbors of macrophage activation |
|  | Neighbors of cell formation |
|  | Neighbors of adaptive immune response |
|  | Neighbors of osteoclast development |
|  | Neighbors of epithelial to mesenchymal transition |
|  | Neighbors of immunity |
|  | Neighbors of cell proliferative response |
|  | Neighbors of chemotaxis |
|  | Neighbors of dendritic cell differentiation |
|  | Neighbors of cell survival |
|  | Neighbors of osteoclast differentiation |
|  | Neighbors of cell phenotype |
|  | Neighbors of T-cell proliferation |
|  | Neighbors of tissue remodeling |
|  | Neighbors of cell adhesion |
|  | Neighbors of T-cell activation |
|  | Neighbors of endothelial cell proliferation |
|  | Neighbors of fibrogenesis |
|  | Neighbors of tumor growth |
|  | Neighbors of dendritic cell development |
|  | Neighbors of tissue repair |
|  | Neighbors of cell count |
|  | Neighbors of cell infiltration |
|  | Neighbors of cell interaction |
|  | Neighbors of SMC proliferation |
|  | Neighbors of lumen formation |
|  | Neighbors of endothelial cell migration |
|  | Neighbors of adipogenesis |
|  | Neighbors of lipid storage |
|  | Neighbors of cell damage |
|  | Neighbors of pregnancy |
|  | Neighbors of monocyte differentiation |
|  | Neighbors of aging |
|  | Neighbors of cell motility |
|  | Neighbors of bone resorption |
|  | Neighbors of monocyte migration |
|  | Neighbors of fibroblast proliferation |
|  | Neighbors of adipocyte differentiation |
|  | Neighbors of macrophage migration |
|  | Neighbors of microglial activation |
|  | Neighbors of neutrophil recruitment |
|  | Neighbors of regeneration |
|  | Neighbors of cell migration |
|  | Neighbors of cancer growth |
|  | Neighbors of stem cell proliferation |
|  | Neighbors of smooth muscle cell migration |
|  | Neighbors of hepatic regeneration |
|  | Neighbors of transendothelial migration |
|  | Neighbors of phagocytosis |
|  | Neighbors of immune system activation |
|  | Neighbors of leukocyte migration |
|  | Neighbors of leukocyte recruitment |
|  | Neighbors of osteoclast formation |
|  | Neighbors of monocyte recruitment |
|  | Neighbors of cell invasion |
|  | Neighbors of heart function |
|  | Neighbors of hemopoiesis |
|  | Neighbors of bone remodeling |
|  | Neighbors of ossification |
|  | Neighbors of T-cell function |
|  | Neighbors of immune system function |
|  | Neighbors of lung development |
|  | Neighbors of autophagy |
|  | Neighbors of monocyte adhesion |
|  | Neighbors of transcription activation |
|  | Neighbors of senescence |
|  | Neighbors of T-cell response |
|  | Neighbors of tumor immunity |
|  | Neighbors of hepatocyte proliferation |
|  | Neighbors of cell transdifferentiation |
|  | Neighbors of neuroprotection |
|  | Neighbors of endothelial cell function |
|  | Neighbors of neuronal death |
|  | Neighbors of cell growth |
|  | Neighbors of ROS generation |
|  | Neighbors of epithelial cell proliferation |
|  | Neighbors of first trimester pregnancy |
|  | Neighbors of cancer cell growth |
|  | Neighbors of sensitization |
|  | Neighbors of hemato-encephalic barrier |
|  | Neighbors of cellular immune response |
|  | Neighbors of cellular senescence |
|  | Neighbors of colony formation |
|  | Neighbors of macrophage differentiation |
|  | Neighbors of cell differentiation |
|  | Neighbors of keratinocyte proliferation |
|  | Neighbors of life span |
|  | Neighbors of cell homeostasis |
|  | Neighbors of psoriasis |
|  | Neighbors of inflammatory disease |
|  | Neighbors of rheumatoid arthritis |
|  | Neighbors of atherosclerosis |
|  | Neighbors of fibrosis |
|  | Neighbors of colitis |
|  | Neighbors of arthritis |
|  | Neighbors of inflammatory bowel disease |
|  | Neighbors of injury |
|  | Neighbors of atherogenesis |
|  | Neighbors of metastasis |
|  | Neighbors of carcinogenesis |
|  | Neighbors of chronic inflammation |
|  | Neighbors of inflammation |
|  | Neighbors of tumor microenvironment |
|  | Neighbors of pathological angiogenesis |
|  | Neighbors of obesity |
|  | Neighbors of vascular disease |
|  | Neighbors of diabetes mellitus |
|  | Neighbors of asthma |
|  | Neighbors of pneumonia |
|  | Neighbors of hyperplasia |
|  | Neighbors of osteoarthritis |
|  | Neighbors of chronic obstructive pulmonary disease |
|  | Neighbors of myocardial infarction |
|  | Neighbors of leukocyte infiltration |
|  | Neighbors of reperfusion injury |
|  | Neighbors of airway inflammation |
|  | Neighbors of hepatitis |
|  | Neighbors of insulin resistance |
|  | Neighbors of infection |
|  | Neighbors of vascular injury |
|  | Neighbors of renal injury |
|  | Neighbors of liver injury |
|  | Neighbors of neuroinflammation |
|  | Neighbors of sepsis |
|  | Neighbors of death |
|  | Neighbors of type 1 diabetes |
|  | Neighbors of encephalomyelitis |
|  | Neighbors of autoimmune disease |
|  | Neighbors of ischemic stroke |
|  | Neighbors of dermatitis |
|  | Neighbors of neutrophil infiltration |
|  | Neighbors of heart disease |
|  | Neighbors of diabetic nephropathy |
|  | Neighbors of lung injury |
|  | Neighbors of immunopathology |
|  | Neighbors of systemic sclerosis |
|  | Neighbors of hepatocellular carcinoma |
|  | Neighbors of autoimmunity |
|  | Neighbors of acute lung injury |
|  | Neighbors of gastric cancer |
|  | Neighbors of experimental autoimmune encephalomyelitis |
|  | Neighbors of colorectal cancer |
|  | Neighbors of cardiac remodeling |
|  | Neighbors of breast cancer |
|  | Neighbors of ischemia |
|  | Neighbors of melanoma |
|  | Neighbors of collagen-induced arthritis |
|  | Neighbors of cartilage degeneration |
|  | Neighbors of neoplasm invasion |
|  | Neighbors of kidney disease |
|  | Neighbors of heart failure |
|  | Neighbors of lung disease |
|  | Neighbors of pulmonary fibrosis |
|  | Neighbors of stroke |
|  | Neighbors of cancer |
|  | Neighbors of type 2 diabetes |
|  | Neighbors of neoplasm |
|  | Neighbors of endometriosis |
|  | Neighbors of vasculitis |
|  | Neighbors of atrophy |
|  | Neighbors of respiratory hypersensitivity |
|  | Neighbors of synovitis |
|  | Neighbors of coronary artery disease |
|  | Neighbors of rupture |
|  | Neighbors of endothelial cell dysfunction |
|  | Neighbors of liver disease |
|  | Neighbors of prostate cancer |
|  | Neighbors of hypertrophy |
|  | Neighbors of osteolysis |
|  | Neighbors of ovarian cancer |
|  | Neighbors of pancreatic cancer |
|  | Neighbors of hypertension |
|  | Neighbors of intimal hyperplasia |
|  | Neighbors of allograft rejection |
|  | Neighbors of proteinuria |
|  | Neighbors of epithelial cell |
|  | Neighbors of keratinocyte |
|  | Neighbors of vascular smooth muscle cell |
|  | Neighbors of umbilical vein endothelial cell |
|  | Neighbors of monocyte |
|  | Neighbors of alveolar macrophage |
|  | Neighbors of synoviocyte |
|  | Neighbors of macrophage |
|  | Neighbors of adipocyte |
|  | Neighbors of activated monocyte |
|  | Neighbors of fibroblast |
|  | Neighbors of airway epithelial cell |
|  | Neighbors of PBMC |
|  | Neighbors of stromal cell |
|  | Neighbors of osteoblast |
|  | Neighbors of pneumocyte |
|  | Neighbors of endometrium cell |
|  | Neighbors of microglia |
|  | Neighbors of intestine epithelium cell |
|  | Neighbors of mononuclear cell |
|  | Neighbors of hepatic stellate cell |
|  | Neighbors of hepatocyte |
|  | Neighbors of kidney mesangial cell |
|  | Neighbors of endothelial cell |
|  | Neighbors of dendritic cell |
|  | Neighbors of Kuppfer cell |
|  | Neighbors of inflammatory cell |
|  | Neighbors of bone marrow macrophage |
|  | Neighbors of peritoneal macrophage |
|  | Neighbors of osteoclast |
|  | Neighbors of neutrophil |
|  | Neighbors of smooth muscle myocyte |
|  | Neighbors of astrocyte |
|  | Neighbors of bone marrow derived dendritic cell |
|  | Neighbors of lung fibroblast |
|  | Neighbors of mesothelial cell |
|  | Neighbors of mononuclear phagocyte |
|  | Neighbors of capillary endothelial cell |
|  | Neighbors of immunocompetent cell |
|  | Neighbors of granulocyte |
|  | Neighbors of cardiac myocyte |
|  | Neighbors of cancer-associated fibroblast |
|  | Neighbors of myeloid cell |
|  | Neighbors of mesenchymal stroma cell |
|  | Neighbors of corneal epithelial cell |
|  | Neighbors of stem cell |
|  | Neighbors of tumor infiltrating macrophage |
|  | Neighbors of sinusoidal cell |
|  | Neighbors of B-cell |
|  | Neighbors of leukocyte |
|  | Neighbors of chondrocyte |
|  | Neighbors of T-cell |
|  | Neighbors of M1 macrophage |
|  | Neighbors of mast cell |
|  | Neighbors of bone marrow stromal cell |
|  | Neighbors of spleen cell |
|  | Neighbors of neuroglia |
|  | Neighbors of thyroid epithelial cell |
|  | Neighbors of hematopoietic cell |
|  | Neighbors of podocyte |
|  | Neighbors of mammary epithelial cell |
|  | Neighbors of conventional dendritic cell |
|  | Neighbors of pericyte |
|  | Neighbors of antigen-presenting cell |
|  | Neighbors of epidermis cell |
|  | Neighbors of phagocyte |
|  | Neighbors of syncytiotrophoblast |
|  | Neighbors of lymphocyte |
|  | Neighbors of Langerhans cell |
|  | Neighbors of thymic epithelial cell |
|  | Neighbors of M2 macrophage |
|  | Neighbors of peritoneum cell |
|  | Neighbors of skin cell |
|  | Neighbors of peripheral lymphocyte |
|  | Neighbors of lung |
|  | Neighbors of liver |
|  | Neighbors of skin |
|  | Neighbors of intestine |
|  | Neighbors of colon |
|  | Neighbors of airway |
|  | Neighbors of blood vessel |
|  | Neighbors of microvessel |
|  | Neighbors of bone |
|  | Neighbors of placenta |
|  | Neighbors of synovial membrane |
|  | Neighbors of uterus |
|  | Neighbors of periodontium |
|  | Neighbors of brain |
|  | Neighbors of breast |
|  | Neighbors of islets of Langerhans |
|  | Neighbors of joint |
|  | Neighbors of kidney glomerulus |
|  | Neighbors of immune system |
|  | Neighbors of spleen |
|  | Neighbors of peritoneum |
|  | Neighbors of allantois |
|  | Neighbors of alveolus |
|  | Neighbors of uterine cervix |
|  | Neighbors of arteriole |
|  | Neighbors of villus |
|  | Neighbors of nose |
|  | Neighbors of eye |
|  | Neighbors of cornea |
|  | Neighbors of hippocampus |
|  | Neighbors of vagina |
|  | Neighbors of thymus gland |
|  | Neighbors of cerebral cortex |
|  | Neighbors of trachea |
|  | Neighbors of NF-kB family |
|  | Neighbors of oxidized LDL |
|  | Neighbors of JNK |
|  | Neighbors of IL1 family |
|  | Neighbors of PPAR |
|  | Neighbors of Jun/Fos |
|  | Neighbors of PKC |
|  | Neighbors of PI3K |
|  | Neighbors of STAT family |
|  | Neighbors of mitogen-activated protein kinase |
|  | Neighbors of IFNAR ligand |
|  | Neighbors of transforming growth factor |
|  | Neighbors of TLR |
|  | Neighbors of cytokine |
|  | Neighbors of interferon |
|  | Neighbors of JAK |
|  | Neighbors of MEK1/2 |
|  | Neighbors of Ras GTPase |
|  | Neighbors of PKA |
|  | Neighbors of ovalbumin |
|  | Neighbors of LDL |
|  | Neighbors of IKBK |
|  | Neighbors of protein tyrosine kinase |
|  | Neighbors of IgG |
|  | Neighbors of AMPK |
|  | Neighbors of LXR |
|  | Neighbors of T-cell receptor |
|  | Neighbors of histone deacetylase |
|  | Neighbors of GPCR |
|  | Neighbors of lectin |
|  | Neighbors of fibrinogen |
|  | Neighbors of aldehyde reductase |
|  | Neighbors of TSH |
|  | Neighbors of NOS |
|  | Neighbors of proteasome endopeptidase complex |
|  | Neighbors of class nuclear receptor with C4 zinc fingers |
|  | Neighbors of PP2B |
|  | Neighbors of AHR subfamily |
|  | Neighbors of ITG |
|  | Neighbors of endostatins |
|  | Neighbors of TORC1 |
|  | Neighbors of histone |
|  | Neighbors of neuropeptides |
|  | Neighbors of caspase |
|  | comon ps\_pos, pprarg\_neg targets |
|  | Model of PPARG signaling in psoriasis |
|  | PPARG negative regulators and targets |
|  | Model of PPARG related pathways in psoriasis (short version) |
|  | sars-cov no binding |
|  | dsRNA induces innate immunity and INFs in alveolar cells |
|  | Dendritic Cell Activation in COVID19 |
|  | 1\_Insufficient activation of immune response in the middle ear epithelium cells in otitis media |
|  | 3\_Role of alveolar macrophages in pulmonary tissue destruction in COPD |
|  | 4\_1\_Polymorphisms associated with inflammatory bowel diseases |
|  | 5\_Human papilloma virus E6 and E7 proteins promote epithelial cell proliferation |
|  | 1\_Microangiopathy in diabetic neuropathy |
|  | drug-target pairs direct regulation |
|  | drug-target moltransport |

---

|  |  |
| --- | --- |
| Group | Genes with Mutations Associated with Systemic Scleroderma |
|  | Genes with Polymorphisms Associated with Migraine |
|  | Genes with Mutations Associated with Endometriosis |
|  | other partners for SARS |

---

|  |  |
| --- | --- |
| Source | curated |
|  | ResNet |
|  | GO component |

---

|  |  |
| --- | --- |
| MedScan ID | 3552 |

---

|  |  |
| --- | --- |
| LocusLink ID | 3552 |
|  | 16175 |
|  | 24493 |
|  | 281250 |

---

|  |  |
| --- | --- |
| Alias | interleukin-1A |
|  | RP23-160G19.8 |
|  | Interleukin-1 large subunit |
|  | preinterleukin 1 alpha |
|  | IL1alpha |
|  | preinterleukin 1 |
|  | hemopoietin 1 |
|  | interleukin IL1A |
|  | IL1F I |
|  | interleukin 1 alpha |
|  | IL1A |
|  | interleukin IL1alpha |
|  | interleukin 1A |
|  | OTTHUMP00000203692 |
|  | interleukin IL1F1 |
|  | interleukin IL1F I |
|  | Hematopoietin I |
|  | Hematopoietin-1 |
|  | preinterleukin I |
|  | OTTMUSP00000016430 |
|  | pro-interleukin-1-alpha |
|  | IL1F1 |
|  | Interleukin-1 alpha subunit |
|  | IL1 |
|  | IL-1A |
|  | IL1-ALPHA |
|  | IL-1 alpha |
|  | interleukin-1 alpha |
|  | IL-1F1 |
|  | interleukin 1, alpha |
|  | IL1A (IL1F1) |
|  | IL1 alpha |

---

|  |  |
| --- | --- |
| GO ID | 0005507 |
|  | 0005125 |
|  | 0005149 |
|  | 0006915 |
|  | 0034605 |
|  | 0002248 |
|  | 0019221 |
|  | 0035234 |
|  | 0097192 |
|  | 0001660 |
|  | 0006955 |
|  | 0070498 |
|  | 0008285 |
|  | 2001240 |
|  | 0045766 |
|  | 0051781 |
|  | 0050715 |
|  | 0010628 |
|  | 0045086 |
|  | 0045840 |
|  | 0050714 |
|  | 0045944 |
|  | 0010575 |
|  | 0046688 |
|  | 0005829 |
|  | 0005576 |
|  | 0005615 |
|  | 0006954 |
|  | 0031424 |
|  | 1904445 |
|  | 0070374 |
|  | 0043123 |
|  | 0046330 |
|  | 0032755 |
|  | 0071639 |
|  | 2000391 |
|  | 1902624 |
|  | 0032308 |
|  | 0010893 |
|  | 0032874 |
|  | 0032956 |
|  | 0051930 |
|  | 0009986 |
|  | 0005737 |
|  | 0005886 |
|  | 0002526 |
|  | 0007507 |
|  | 0010469 |
|  | 0033591 |
|  | 0010332 |
|  | 0009408 |
|  | 0001666 |
|  | 0032496 |
|  | 0014070 |
|  | 0010033 |
|  | 0010243 |
|  | 0010193 |
|  | 0007283 |
|  | 0042060 |
|  | 0006916 |
|  | 0002437 |
|  | 0043066 |
|  | 0005515 |
|  | 0005150 |
|  | 0008283 |
|  | 0008083 |
|  | 0004871 |
|  | 0007267 |
|  | 0006935 |
|  | 0000074 |

---

|  |  |
| --- | --- |
| KEGG ID | hsa:3552 |
|  | mmu:16175 |
|  | rno:24493 |

---

|  |  |
| --- | --- |
| MedLine Reference | 10432220 |
|  | 12193539 |
|  | 12270120 |
|  | 12438087 |
|  | 12479028 |
|  | 12534936 |
|  | 12785009 |
|  | 12878449 |
|  | 14643175 |
|  | 15025948 |
|  | 15032628 |
|  | 15063785 |
|  | 15154090 |
|  | 15467353 |
|  | 15671867 |
|  | 15990800 |
|  | 16083355 |
|  | 16616101 |
|  | 16825798 |
|  | 16905534 |
|  | 17117141 |
|  | 2471704 |
|  | 10226804 |
|  | 10349636 |
|  | 10452974 |
|  | 10622417 |
|  | 10651053 |
|  | 10744718 |
|  | 10830786 |
|  | 11042159 |
|  | 11076861 |
|  | 11217851 |
|  | 11342576 |
|  | 11518254 |
|  | 11536012 |
|  | 11698278 |
|  | 11717188 |
|  | 11754748 |
|  | 11773602 |
|  | 11809690 |
|  | 11840457 |
|  | 11871152 |
|  | 11883745 |
|  | 11899425 |
|  | 11959104 |
|  | 11988319 |
|  | 11991723 |
|  | 12010793 |
|  | 12012430 |
|  | 12070778 |
|  | 12102659 |
|  | 12112371 |
|  | 12151598 |
|  | 12193704 |
|  | 12270723 |
|  | 12466851 |
|  | 12477932 |
|  | 12598651 |
|  | 12663678 |
|  | 12799211 |
|  | 1284644 |
|  | 12975454 |
|  | 1349539 |
|  | 1353383 |
|  | 1362103 |
|  | 1371115 |
|  | 1421760 |
|  | 14612453 |
|  | 14662844 |
|  | 14744933 |
|  | 14966082 |
|  | 15232066 |
|  | 15271886 |
|  | 1531270 |
|  | 15314695 |
|  | 15331419 |
|  | 1533388 |
|  | 15381720 |
|  | 1552283 |
|  | 1552482 |
|  | 15549727 |
|  | 15591249 |
|  | 15613243 |
|  | 15684062 |
|  | 1572101 |
|  | 1577194 |
|  | 15922486 |
|  | 16026578 |
|  | 16046471 |
|  | 1611993 |
|  | 16137777 |
|  | 16141072 |
|  | 16141073 |
|  | 16256210 |
|  | 16415102 |
|  | 1649220 |
|  | 16517748 |
|  | 16565512 |
|  | 16567518 |
|  | 16569679 |
|  | 1672162 |
|  | 16825293 |
|  | 1685143 |
|  | 1686838 |
|  | 16936270 |
|  | 1828262 |
|  | 1828896 |
|  | 1970329 |
|  | 1970330 |
|  | 2166945 |
|  | 2267629 |
|  | 2419912 |
|  | 2466396 |
|  | 2613234 |
|  | 2759422 |
|  | 2889668 |
|  | 3022289 |
|  | 3149717 |
|  | 6209582 |
|  | 7476020 |
|  | 7539003 |
|  | 7579389 |
|  | 7752808 |
|  | 7819439 |
|  | 7826951 |
|  | 7848516 |
|  | 7873879 |
|  | 7902327 |
|  | 7958862 |
|  | 7959775 |
|  | 8001745 |
|  | 8012118 |
|  | 8027056 |
|  | 8031998 |
|  | 8138038 |
|  | 8177505 |
|  | 8207122 |
|  | 8207237 |
|  | 8224251 |
|  | 8231109 |
|  | 8260598 |
|  | 8268655 |
|  | 8288218 |
|  | 8288233 |
|  | 8306498 |
|  | 8327661 |
|  | 8352883 |
|  | 8432536 |
|  | 8449520 |
|  | 8462855 |
|  | 8464030 |
|  | 8482578 |
|  | 8524866 |
|  | 8543912 |
|  | 8578682 |
|  | 8630372 |
|  | 8699818 |
|  | 8751373 |
|  | 8770556 |
|  | 8798439 |
|  | 8816919 |
|  | 8818542 |
|  | 8833235 |
|  | 8838580 |
|  | 8854867 |
|  | 8881659 |
|  | 8920166 |
|  | 8932996 |
|  | 8953518 |
|  | 9344502 |
|  | 9565638 |
|  | 9809553 |
|  | 9854157 |
|  | 9886396 |
|  | 10441334 |
|  | 10512743 |
|  | 10625660 |
|  | 10748004 |
|  | 10860666 |
|  | 10861041 |
|  | 10953177 |
|  | 10998424 |
|  | 11093146 |
|  | 11145836 |
|  | 11278614 |
|  | 11448121 |
|  | 11466363 |
|  | 11554782 |
|  | 11574262 |
|  | 11774556 |
|  | 11774564 |
|  | 11940570 |
|  | 11991668 |
|  | 11991722 |
|  | 12037600 |
|  | 12047332 |
|  | 12051868 |
|  | 12101079 |
|  | 12115182 |
|  | 12149413 |
|  | 12209090 |
|  | 12220547 |
|  | 12242547 |
|  | 12362317 |
|  | 12370389 |
|  | 12445604 |
|  | 12453471 |
|  | 12519748 |
|  | 12528118 |
|  | 12528119 |
|  | 12530978 |
|  | 12613995 |
|  | 12631337 |
|  | 12651617 |
|  | 12667716 |
|  | 12673844 |
|  | 12710950 |
|  | 12714264 |
|  | 12742380 |
|  | 12746488 |
|  | 12913118 |
|  | 12928381 |
|  | 12930304 |
|  | 12931024 |
|  | 12946945 |
|  | 13679381 |
|  | 14633625 |
|  | 14674121 |
|  | 14688369 |
|  | 14705223 |
|  | 14983027 |
|  | 15007345 |
|  | 15009111 |
|  | 15036245 |
|  | 15039285 |
|  | 15082121 |
|  | 15117956 |
|  | 15130917 |
|  | 15381245 |
|  | 15468358 |
|  | 15489227 |
|  | 15514971 |
|  | 15563458 |
|  | 15587751 |
|  | 15638425 |
|  | 15679580 |
|  | 15732864 |
|  | 15737206 |
|  | 15745939 |
|  | 1584804 |
|  | 15951664 |
|  | 15954918 |
|  | 15974847 |
|  | 15986200 |
|  | 16101131 |
|  | 16246569 |
|  | 16291395 |
|  | 16318580 |
|  | 16378839 |
|  | 16410064 |
|  | 16410066 |
|  | 16428739 |
|  | 16433908 |
|  | 16478776 |
|  | 16504015 |
|  | 16564702 |
|  | 16564703 |
|  | 16619041 |
|  | 16684536 |
|  | 1675504 |
|  | 16788102 |
|  | 16879223 |
|  | 16888077 |
|  | 16945028 |
|  | 16971486 |
|  | 16978691 |
|  | 17012236 |
|  | 1924307 |
|  | 2635664 |
|  | 2783369 |
|  | 2989698 |
|  | 2994016 |
|  | 3485152 |
|  | 3486405 |
|  | 3493774 |
|  | 7494249 |
|  | 7499244 |
|  | 7526541 |
|  | 7566121 |
|  | 7695626 |
|  | 7818827 |
|  | 7857652 |
|  | 7878046 |
|  | 8188271 |
|  | 8370968 |
|  | 8436814 |
|  | 8621446 |
|  | 9169134 |
|  | 9820540 |
|  | 15253926 |
|  | 17174540 |
|  | 3261832 |
|  | 3262865 |
|  | 15489334 |
|  | 16636934 |
|  | 16965825 |
|  | 17197697 |
|  | 17205326 |
|  | 17244613 |
|  | 17290104 |
|  | 17293495 |
|  | 17309781 |
|  | 17335371 |
|  | 17335373 |
|  | 17360358 |
|  | 17371964 |
|  | 17378887 |
|  | 17440718 |
|  | 17449468 |
|  | 17460270 |
|  | 17473736 |
|  | 17541168 |
|  | 17621543 |
|  | 2346741 |
|  | 3281727 |
|  | 8346241 |
|  | 11418636 |
|  | 11466424 |
|  | 12878687 |
|  | 14634130 |
|  | 17277081 |
|  | 17283139 |
|  | 17307724 |
|  | 17609389 |
|  | 3126184 |
|  | 17610660 |
|  | 17548042 |

---

|  |  |
| --- | --- |
| Organism | Homo sapiens {Organism urn:agi-taxid:9606} |
|  | Mus musculus {Organism urn:agi-taxid:10090} |
|  | Rattus norvegicus {Organism urn:agi-taxid:10116} |
|  | Homo sapiens |
|  | Mus musculus |
|  | Rattus norvegicus |
|  | Bos taurus |

---

|  |  |
| --- | --- |
| Mouse chromosome position | 2 62.9 cM |
|  | 2 73.0 cM |

---

|  |  |
| --- | --- |
| OMIM ID | 147760 |

---

|  |  |
| --- | --- |
| Rat chromosome position | 3q36 |

---

|  |  |
| --- | --- |
| Hugo ID | 5991 |
|  | HGNC:5991 |

---

|  |  |
| --- | --- |
| Human chromosome position | 2q14.1 |
|  | 2q14 |

---

|  |  |
| --- | --- |
| Swiss-Prot Accession | P01583 |
|  | P01583.1 |
|  | P01582 |
|  | Q3U0Y6 |
|  | P01582.1 |
|  | P16598 |
|  | P16598.1 |
|  | Q53QF9 |
|  | Q7RU02 |
|  | Q546R9 |
|  | Q62161 |
|  | Q71VD0 |
|  | Q9ESW2 |
|  | IL1A\_HUMAN |
|  | IL1A\_MOUSE |
|  | IL1A\_RAT |
|  | Q53QF9\_HUMAN |
|  | Q3U0Y6\_MOUSE |

---

|  |  |
| --- | --- |
| PIR ID | A23385 |
|  | A01846 |
|  | JX0064 |

---

|  |  |
| --- | --- |
| GenBank ID | NC\_000002 |
|  | NM\_000575 |
|  | NP\_000566 |
|  | NG\_008850 |
|  | AC079753 |
|  | AC112235 |
|  | AAX93054 |
|  | AF536338 |
|  | AAM96189 |
|  | BN000002 |
|  | CAD29871 |
|  | CH471217 |
|  | EAW73604 |
|  | X03833 |
|  | CAA27448 |
|  | AA856674 |
|  | AK314850 |
|  | BAG37367 |
|  | BC013142 |
|  | AAH13142 |
|  | BC035169 |
|  | BT007014 |
|  | AAP35660 |
|  | CA307202 |
|  | CR457414 |
|  | CAG33695 |
|  | M15329 |
|  | AAA59133 |
|  | X02531 |
|  | CAA26371 |
|  | X02851 |
|  | CAA26604 |
|  | X56086 |
|  | CAA39566 |
|  | P01583 |
|  | NC\_000068 |
|  | XM\_006498793 |
|  | XP\_006498856 |
|  | NM\_010554 |
|  | NP\_034684 |
|  | XM\_006498794 |
|  | XP\_006498857 |
|  | AF010237 |
|  | AAC28999 |
|  | AL772347 |
|  | AY902318 |
|  | AAX90603 |
|  | CH466519 |
|  | EDL28236 |
|  | EDL28237 |
|  | AK156449 |
|  | BAE33715 |
|  | BC003727 |
|  | AAH03727 |
|  | M14639 |
|  | AAA40058 |
|  | X01450 |
|  | CAA25682 |
|  | P01582 |
|  | NC\_005102 |
|  | NM\_017019 |
|  | NP\_058715 |
|  | AC\_000071 |
|  | AABR07053712 |
|  | AABR07053713 |
|  | AABR07072926 |
|  | AAHX01025007 |
|  | AAHX01025008 |
|  | AF016188 |
|  | AAD01583 |
|  | CH473949 |
|  | EDL80161 |
|  | EDL80162 |
|  | AJ245642 |
|  | CAC03995 |
|  | AJ245643 |
|  | CAC03996 |
|  | D00403 |
|  | BAA00306 |
|  | KF027366 |
|  | AHW98144 |
|  | P16598 |
|  | ABM84782 |
|  | AAHY01020659 |
|  | ABM81601 |
|  | DQ893856 |
|  | AMYH02004593 |
|  | AC\_000024 |
|  | AABR06026122 |
|  | AABR06026123 |
|  | XP\_006498855 |
|  | AC\_000134 |
|  | NC\_018913 |
|  | DQ890675 |
|  | ABBA01074249 |
|  | CAM15804 |
|  | XM\_006498792 |
|  | NT\_022135 |
|  | NW\_001838841 |
|  | NW\_921474 |
|  | AC\_000045 |
|  | Q53QF9 |
|  | NT\_039207 |
|  | NW\_001030694 |
|  | Q3U0Y6 |
|  | Q62161 |
|  | NW\_047658 |
|  | NW\_001084813 |
|  | Q71VD0 |
|  | Q9ESW2 |
|  | Q546R9 |
|  | NW\_000178 |
|  | 124301 |
|  | 220783 |
|  | 4102785 |
|  | 81870927 |
|  | 81872523 |
|  | 81887923 |
|  | 9844629 |
|  | 9844631 |
|  | 123796899 |
|  | 124298 |
|  | 13277632 |
|  | 200757 |
|  | 3282326 |
|  | 52669 |
|  | 62635466 |
|  | 74224223 |
|  | 81863067 |
|  | 119594010 |
|  | 123994361 |
|  | 124297 |
|  | 15341913 |
|  | 186278 |
|  | 22385324 |
|  | 28317371 |
|  | 30582867 |
|  | 33786 |
|  | 33795 |
|  | 35661 |
|  | 48146945 |
|  | 62702127 |
|  | 74740635 |
|  | 755744 |
|  | NT\_086624 |
|  | 124296 |
|  | 163199 |
|  | 163227 |
|  | 445 |
|  | AAA30583 |
|  | AAA30590 |
|  | CAA31017 |
|  | M36182 |
|  | M37210 |
|  | NC\_007309 |
|  | NM\_174092 |
|  | NP\_776517 |
|  | NW\_001492942 |
|  | P08831 |
|  | X12497 |
|  | 123979544 |
|  | 123857683 |
|  | 148696289 |
|  | 148696290 |
|  | 149023267 |
|  | 149023268 |

---

|  |  |
| --- | --- |
| Swiss-Prot ID | IL1A\_HUMAN |
|  | IL1A\_MOUSE |
|  | IL1A\_RAT |
|  | Q3U0Y6\_MOUSE |
|  | Q53QF9\_HUMAN |

---

|  |  |
| --- | --- |
| Cell Localization | Secreted |
|  | Extracellular |

---

|  |  |
| --- | --- |
| Ensembl ID | ENSG00000115008 |
|  | ENSP00000263339.3 |
|  | ENST00000263339.3 |
|  | ENSMUSG00000027399 |
|  | ENSMUSP00000028882.1 |
|  | ENSMUST00000028882.1 |
|  | ENSRNOG00000004575 |
|  | ENSRNOP00000071252.1 |
|  | ENSRNOT00000080818.1 |
|  | ENSP00000263339 |
|  | ENST00000263339 |
|  | ENSMUST00000028882 |
|  | ENSRNOT00000080818 |
|  | ENSRNOP00000071252 |
|  | ENSMUSP00000028882 |

---

|  |  |
| --- | --- |
| MGI ID | MGI:96542 |
|  | 96542 |

---

|  |  |
| --- | --- |
| RGD ID | 2890 |

---

|  |  |
| --- | --- |
| TCDB ID | 1.A.109.1.1 |

---

|  |  |
| --- | --- |
| Unigene ID | Hs.1722 |
|  | Rn.12300 |
|  | Mm.15534 |
|  | Bt.191 |

---

|  |  |
| --- | --- |
| Homologene ID | 480 |

---

|  |  |
| --- | --- |
| Shape | Rhomb |

---

|  |  |
| --- | --- |
| IPI ID | IPI00136895 |
|  | IPI00213945 |
|  | IPI00003096 |
|  | IPI00192438 |
|  | IPI00781873 |

---

|  |  |
| --- | --- |
| Microarray ID | 94755\_at |

---

|  |  |
| --- | --- |
| Homo sapiens Chromosome position | 2q14 |

---

|  |  |
| --- | --- |
| Ensembl Transcript ID | ENST00000263339 |
|  | ENST00000409071 |

---

|  |  |
| --- | --- |
| FunctionalClass | interleukin-1, Type I receptor binding |
|  | signal transducer |
|  | cytokine |
|  | growth factor |
|  | interleukin-1 receptor binding |
|  | protein binding |

---

|  |  |
| --- | --- |
| Bos taurus Chromosome position | 11 |

---

|  |  |
| --- | --- |
| Mus musculus Chromosome position | 2 73.0 cM |

---

|  |  |
| --- | --- |
| KEGG pathway | Hematopoietic cell lineage |
|  | Type I diabetes mellitus |
|  | Apoptosis |
|  | Cytokine-cytokine receptor interaction |
|  | MAPK signaling pathway |

---

|  |  |
| --- | --- |
| Rattus norvegicus Chromosome position | 3q36 |

---

|  |  |
| --- | --- |
| Ensembl Gene ID | ENSG00000115008 |

---

|  |  |
| --- | --- |
| LocatorString | digtext:C:\Documents and Settings\yuryev\My Documents\Customers\Kristina Hettne\CPRS input entities.txt |
|  | pmquery:invasive+breast+cancer |

---
